# Osteogenesis imperfecta: a cross-sectional study of skeletal and extraskeletal features in a large cohort of Italian patients

**DOI:** 10.3389/fendo.2023.1299232

**Published:** 2024-01-11

**Authors:** Marina Mordenti, Manila Boarini, Federico Banchelli, Diego Antonioli, Serena Corsini, Maria Gnoli, Manuela Locatelli, Elena Pedrini, Eric Staals, Giovanni Trisolino, Marcella Lanza, Luca Sangiorgi

**Affiliations:** ^1^ Department of Rare Skeletal Disorders, IRCCS Istituto Ortopedico Rizzoli, Bologna, Italy; ^2^ Unit of Pediatrics Orthopedics and Traumatology, IRCCS Istituto Ortopedico Rizzoli, Bologna, Italy; ^3^ 3^rd^Orthopedic and Traumatological Clinic Prevalently Oncologic, IRCCS Istituto Ortopedico Rizzoli, Bologna, Italy

**Keywords:** osteogenesis imperfecta, phenotype, extraskeletal manifestations, bone fragility, brittle bone disease, collagen

## Abstract

**Introduction:**

The present study aims to describe a large cohort of Italian patients affected by osteogenesis imperfecta, providing a picture of the clinical bony and non-bony features and the molecular background to improve knowledge of the disease to inform appropriate management in clinical practice.

**Methods:**

A total of 568 subjects (from 446 unrelated Italian families) affected by osteogenesis imperfecta who received outpatient care at Istituto Ortopedico Rizzoli from 2006 to 2021 were considered in the present study.

**Results:**

Skeletal and extraskeletal features were analyzed showing a lower height (mean z-scores equal to -1.54 for male patients and -1.47 for female patients) compared with the general Italian population. Half of the patient population showed one or more deformities, and most of the patients had suffered a relatively low number of fractures (<10). An alteration in the sclera color was identified in 447 patients. Similarly, several extraskeletal features, like deafness, dental abnormalities, and cardiac problems, were investigated. Additionally, inheritance and genetic background were evaluated, showing that most of the patients have a positive family history and the majority of pathogenic variants detected were on collagen genes, as per literature.

**Conclusion:**

This study supports the definition of a clear picture of the heterogeneous clinical manifestations leading to variable severity in terms of skeletal and extra-skeletal traits and of the genetic background of an Italian population of osteogenesis imperfecta patients. In this perspective, this clearly highlights the crucial role of standardized and structured collection of high-quality data in disease registries particularly in rare disease scenarios, helping clinicians in disease monitoring and follow-up to improve clinical practice.

## 1 Introduction

Osteogenesis imperfecta (OI—MIM#166200, MIM#166210, MIM#259420, MIM#166220), also known as “brittle bone disease,” is a rare hereditary disorder of the bones with a prevalence at birth of approximately 5–10 in 100,000 live births (1, 2). Key signs of the disease are recurrent fractures, bony deformities, and short stature due to bone fragility. Considering the presence of a number of extraskeletal features, like joint hyperlaxity, colored sclerae, dentinogenesis imperfecta (DI), and hearing impairments, the care of OI patients requires a multidisciplinary approach (1–3). The presentation of OI is characterized by a wide variability of clinical manifestations ranging from mild signs to severe forms. In 1979, Sillence proposed a classification based on clinical and radiographic findings (4). The most frequent and mildest form of OI is type I defined by bone fragility, no evident deformities, and blue sclerae. OI type II is the lethal perinatal type, representing the most severe form with multiple fractures during intrauterine life or at birth. OI type III patients are characterized by fractures, progressive bone deformities, normal sclerae, and frequently DI. Patients with OI type IV present an intermediate severity between type I and III with progressive deformities of the limbs and spine. In 2014, this classification was revised with the introduction of the OI type V, characterized by interosseous ossification and propensity to develop hyperplastic callus (5, 6).

The genetic background is highly heterogeneous, with both autosomal dominant and recessive inheritance. The majority of OI patients harbor dominant variants in *COL1A1* (MIM#120150) or *COL1A2* (MIM#120160) genes, which encode, respectively, alpha 1 and alpha 2 chains of type I collagen (7), the most abundant matrix protein of the human body (8). Hitherto, a large group of non-collagen genes has been also described, mainly causing autosomal recessive OI (1, 9, 10). Those genes are key elements of pathways related to bone mineralization, collagen modification, and processing and differentiation of osteoblasts (11).

The objective of the present study is to accurately describe a large cohort of Italian patients affected by osteogenesis imperfecta seen during a period of 16 years, providing a representative picture of the phenotypic presentation and the molecular characteristics in order to improve knowledge of disease variability for appropriate management in clinical practice.

## 2 Materials and methods

### 2.1 Design, setting, and participants

We carried out a cross-sectional study aiming to present an epidemiological picture in a large cohort of Italian patients with OI who received outpatient care at Istituto Ortopedico Rizzoli (IOR) in Bologna, Italy, from 2006 to 2021. IOR is a referral center highly specialized in rare skeletal disorders and, since 2017, has been the coordinator of the European Reference Network on Rare Bone Disorders (ERN BOND). Data captured from electronic health records, patients’ reports, and medical files were stored in a GDPR-compliant IT platform, serving as the basis for the Registry of Osteogenesis Imperfecta (ROI—NCT04115774).

The inclusion criteria were all consecutive male and female patients of all ages with a confirmed clinical diagnosis of osteogenesis imperfecta (6), seen from 2006 to 2021, and the presence of a detailed dataset that includes anthropometric information and description of skeletal features and extraskeletal traits. In addition, genetic data and inheritance information were included. Patients with missing data were excluded. Whenever possible, patients were classified according to the Sillence classification in five types by expert orthopedicians and clinicians, depending on phenotypic features and inheritance patterns (6). For the entire series, data were referred to the last available visit with complete data.

The exclusion criteria were all bone fragilities other than osteogenesis imperfecta and OI type II due to incomplete or insufficient data.

The present study was approved by the Area Vasta Emilia Centro Ethics Committee (CE-AVEC) in February 2021 (1093/2020/Sper/IOR).

### 2.2 Data collection and clinical features

Clinical data were collected according to a standardized protocol that comprised patient demographics (including gender and age); anthropometric parameters, in terms of height, weight (percentile and *z*-score) (12), and body mass index (BMI) [percentile, *z*-score, and World Health Organization (WHO) classes]; and skeletal features including a range of fractures (0, 1–4, 5–10, 11–20, 21–30, >30), deformities and limitation details (presence/absence, number, localization grouped according to macrosites), vertebral collapse, facial dysmorphisms (triangular face, maxilla deformity, frontal bossing, prognathism, and concave nasal ridge), and bone densitometry (BMD), described as normal, osteopenia, and osteoporosis (osteoporosis: >−2.5; osteopenia: between −1.0 and −2.5; normal: >−1.0; calculated according to Rossini et al., 2016) (13) captured from descriptive reports assessed by dual-energy X-ray absorptiometry either at the lumbar or femoral site. Extraskeletal traits consisted of skin abnormalities (presence/absence and details), deafness (presence/absence and details), cardiac defects (aortic, mitral, tricuspid, pulmonary valvulopathies), color of sclerae (white, blue, gray/purple), joint hyperlaxity (JH), dentinogenesis imperfecta, and other dentition defects. In addition, genetic and familial information were captured, including presence/absence, involved gene, type and significance of the variant, mutation effect (quantitative/haploinsufficiency, qualitative/structural), and inheritance.

Age- and sex-adjusted percentiles and *z*-scores for height, weight, and BMI were calculated by using different criteria based on the age and gender of the subjects. For patients aged 2–20 years, the reference charts for the Italian population were used (12), whereas for individuals under 2 years of age, the reference charts were those of the WHO. For patients aged more than 20 years, the values for 20-year-old subjects were considered, based on the reference charts for the Italian population (12). Each adult subject was classified into one of the following BMI classes: very underweight (BMI < 16), underweight (BMI ≥16 and <18.5), normal weight (BMI ≥18.5 and <25), overweight (BMI ≥25 and <30), and obese (BMI ≥30). For patients aged less than 20 years, BMI classes were assigned by using the method proposed by Cole et al. (14) based on reference charts for the Italian population (12).

### 2.3 Analyses of causative variants in OI-related genes

When biological material (blood, saliva, DNA) was available, genetic analyses were performed to identify pathogenic variants of OI causative genes. Genetic analysis of *COL1A1* and *COL1A2*, evaluating exons and exon–intron boundaries, was performed by direct sequencing and/or target sequencing. Large rearrangements were assessed by multiple ligation-dependent probe amplification and/or quantitative polymerase chain reaction. Recessive genes and *IFITM5* were analyzed based on genetic knowledge at sample collection and clinical evaluation.

### 2.4 Statistical analyses

Descriptive characteristics of the patients were reported as the mean ± standard deviation (SD) for quantitative variables or as absolute and percentage numbers for categorical variables. Prevalence measures were expressed as the percentage of patients with specific characteristics, with a two-sided 95% confidence interval (CI) based on the binomial distribution Wilson score method. Statistical analyses were carried out using R 3.6.3 statistical software (The R Foundation for Statistical Computing, Wien).

## 3 Results

### 3.1 Demographics, anthropometrics, and OI types

A total of 568 subjects with continuous follow-up—from 446 unrelated Italian families—were included. According to the Van Dijk classification (6), patients were classified into type I (54.6%), type III (5.5%), type IV (10.4%), and type V (0.4%). The class could not be assigned to 29% of the patients due to atypical manifestations (borderline aspects between types) and the very young age of the patients. There was almost equal distribution between female (53.5%) and male patients. Patients covered an age range from 0 to 75 years (mean age: 22.2 years) and 50% of them belong to infant (0–1), children (2–9), and adolescent groups (10–17) (see **Table 1**).

**Table 1:** Demographic and anthropometric characteristics of OI patients.

			All patients (*n* = 568)	
OI type	*Unknown*	*n* %	166	29.2%
	*I*	*n* %	310	54.6%
	*III*	*n* %	31	5.5%
	*IV*	*n* %	59	10.4%
	*V*	*n* %	2	0.4%
Gender	*Male*	*n* %	264	46.5%
Age	*Years*	Mean SD	22.2	18.1
	*0–1*	*n* %	63	11.1%
	*2–9*	*n* %	135	23.8%
	*10–17*	*n* %	88	15.5%
	*18–34*	*n* %	134	23.6%
	*35–50*	*n* %	100	17.6%
	*50–65*	*n* %	35	6.2%
	*65–75*	*n* %	13	2.3%
Height^a^	*z-score*	Mean SD	−1.50	2.21
	*Percentile*	Mean SD	25.5	29.9
Weight^b^	*z-score*	Mean SD	−0.63	1.76
	*Percentile*	Mean SD	38.9	33.3
BMI^c^	*z-score*	Mean SD	0.18	1.49
	*Percentile*	Mean SD	55.6	33.6
	*Very underweight*	*n* %	10	2.3%
	*Underweight*	*n* %	54	9.5%
	*Normal weight*	*n* %	261	46.0%
	*Overweight*	*n* %	72	12.7%
	*Obese*	*n* %	46	8.1%

SD, standard deviation.

a Data were missing for 79 patients (13.9%).

b Data were missing for 120 patients (21.1%).

c Data were missing for 125 patients (22%).

The height ranged from 49 to 186 cm in male patients and from 43 to 175 cm in female patients with mean *z*-scores equal to −1.54 and −1.47 compared with the general Italian population (**Supplementary Table S1**). In detail, the OI type I group showed a *z*-score equal to −0.89, while types III, IV, and V present *z*-scores equal to −4.89, −2.77, and −2.74, respectively (**Supplementary Table S2**). The weight range was 3-114 kg, z-score: -0.60 and range 2 – 95 kg, z-score: -0.66 for male and female patients, respectively. BMI showed that most of the patients are of normal weight (58.9%), even if overweight and obese patients represented almost 26.7%, while underweight and very underweight patients represented 14.5% of the entire cohort, taking into consideration that data pertaining to 125 patients were missing. The BMI behaved similarly considering male and female patients separately (**Supplementary Table S1**). Subdividing patients according to OI types, the normal weight BMI was less frequent in types III and IV (45.5% and 40.8%, respectively) with an increment in all the other BMI classes (**Supplementary Table S2**).

### 3.2 Skeletal features

#### 3.2.1 Deformities and limitations

OI is frequently characterized by bone deformity and functional limitation, due to altered bone structure and/or as a result of fracture(s). Half of the cohort showed one or more deformities. Most of them involved the trunk (259 subjects, 45.6%, 40.9% in male patients and 49.7% in female patients)—mainly impairing the spine alignment, in terms of scoliosis, kyphosis, and lordosis (162, 54, and 17 subjects, respectively), followed by lower limb (88 subjects, 15.5%) and upper limb deformities (33 subjects, 5.8%). A similar trend was observed when analyzing male and female patients separately (**Supplementary Tables S3, S4**), while types III, IV, and V showed higher presence of trunk involvement (83.9%, 67.8%, and 100%, respectively) (**Supplementary Tables S5–S8**). Functional limitations were detected in 15 patients out of 568 (2.7%), affecting upper or lower long bones and the pelvis (see **Table 2**).

**Table 2:** Prevalence of deformities and limitations.

Anatomical site	All patients (*n* = 568)								
	Deformity			Limitation			Deformity or limitation		
	*n*	%	95% CI	*n*	%	95% CI	*n*	%	95% CI
Any deformity or limitation	286	50.4%	46.3%–54.4%	15	2.6%	1.6%–4.3%	301	53.0%	48.9%–57.1%
Head and neck	2	0.4%	0.1%–1.3%	0	0.0%		2	0.4%	0.1%–1.3%
Upper limbs	33	5.8%	4.2%–8.0%	6	1.1%	0.5%–2.3%	39	6.9%	5.1%–9.2%
Arm	2	0.4%	0.1%–1.3%	0	0.0%		2	0.4%	0.1%–1.3%
Elbow	9	1.6%	0.8%–3.0%	4	0.7%	0.3%–1.8%	13	2.3%	1.3%–3.9%
Forearm	6	1.1%	0.5%–2.3%	1	0.2%	0.0%–1.0%	7	1.2%	0.6%–2.5%
Hand	20	3.5%	2.3%–5.4%	0	0.0%		20	3.5%	2.3%–5.4%
Shoulder	11	1.9%	1.1%–3.4%	1	0.2%	0.0%–1.0%	12	2.1%	1.2%–3.7%
Lower limbs	88	15.5%	12.7%–18.7%	11	1.9%	1.1%–3.4%	99	17.4%	14.5%–20.8%
Ankle	5	0.9%	0.4%–2.0%	1	0.2%	0.0%–1.0%	6	1.1%	0.5%–2.3%
Foot	41	7.2%	5.4%–9.6%	6	1.1%	0.5%–2.3%	47	8.3%	6.3%–10.8%
Knee	37	6.5%	4.8%–8.8%	3	0.5%	0.2%–1.5%	40	7.0%	5.2%–9.4%
Leg	31	5.5%	3.9%–7.6%	2	0.4%	0.1%–1.3%	33	5.8%	4.2%–8.0%
Thigh	30	5.3%	3.7%–7.4%	1	0.2%	0.0%–1.0%	31	5.5%	3.9%–7.6%
Trunk	259	45.6%	41.5%–49.7%	0	0.0%		259	45.6%	41.5%–49.7%
Ribs	63	11.1%	8.8%–13.9%	0	0.0%		63	11.1%	8.8%–13.9%
Spine alignment	233	41.0%	37.0%–45.1%	0	0.0%		233	41.0%	37.0%–45.1%
Lordosis	17	3.0%	1.9%–4.7%	0	0.0%		17	3.0%	1.9%–4.7%
Kyphosis	54	9.5%	7.4%–12.2%	0	0.0%		54	9.5%	7.4%–12.2%
Scoliosis	162	28.5%	25.0%–32.4%	0	0.0%		162	28.5%	25.0%–32.4%
Other spine problems	16	2.8%	1.7%–4.5%	0	0.0%		16	2.8%	1.7%–4.5%
Pelvis	15	2.6%	1.6%–4.3%	8	1.4%	0.7%–2.8%	23	4.0%	2.7%–6.0%

CI, confidence interval.

#### 3.2.2 Clinical features

A typical manifestation of OI is the presence of multiple fractures. At inclusion in the study, most patients (71.3%) had suffered a relatively low number of fractures (1–4 and 5–10), although 16 patients reported more than 30 fractures in their medical history. Analysis considering male and female patients separately showed data in line with the entire cohort (**Supplementary Table S9**). Vertebral compression fractures were observed in 10 patients.

The presence of osteopenia or osteoporosis was identified in approximately 40% of the cohort (79 and 145 patients, respectively)—also considering male and female patients separately—and a higher presence of osteoporosis in types III–IV (see **Supplementary Tables S10**, **S11**). One-hundred seven patients (21%) manifested facial dysmorphisms, such as frontal bossing, maxilla dysmorphism, and triangular face. Detailed information is listed in **Table 3**.

**Table 3:** Prevalence of skeletal features.

		All patients (*n* = 568)		
		*n*	%	95% CI
Number of fractures	*0*	73	12.9%	10.3%–15.9%
	*1–4*	234	41.2%	37.2%–45.3%
	*5–10*	171	30.1%	26.5%–34.0%
	*11–20*	56	9.9%	7.7%–12.6%
	*21–30*	18	3.2%	2.0%–5.0%
	*>30*	16	2.8%	1.7%–4.5%
Bone densitometry	*Osteopenia*	79	13.9%	11.3%–17.0%
	*Osteoporosis*	145	25.5%	22.1%–29.3%
Any facial dysmorphism	*Yes*	117	20.6%	17.5%–24.1%
Triangular face	*Yes*	32	5.6%	4.0%–7.8%
Maxillodysmorphism	*Yes*	42	7.4%	5.5%–9.8%
Frontal bossing	*Yes*	60	10.6%	8.3%–13.4%
Other facial dysmorphism	*Yes*	5	0.9%	0.4%–2.0%
Vertebral compression fractures	*Yes*	10	1.8%	1.0%–3.2%

CI, confidence interval.

### 3.3 Prevalence of extraskeletal features

Collagen is known to be an important component of organs and systems other than bones, so non-skeletal manifestations are frequently present in OI patients.

Alteration of the color of the sclera was identified in approximately 80% of the patients (447 cases), with a higher presence of bluish sclera over the gray/purple discoloration (65% vs. 13.9%), with a slightly higher prevalence in female than in male patients (83.1% and 74.2%, respectively). In addition, colored sclerae were more present in OI type I (85.1%) in comparison with OI type III (61.3%) (see **Supplementary Tables S12–S14**).

Eighty-five patients (15%) presented abnormalities of the skin, most of them presenting loose skin. Hearing impairment was present in 97 patients (17.1%) with an almost equal distribution among conductive, sensorineural, and mixed deafness (see **Table 4**).

**Table 4:** Prevalence of extraskeletal features.

		All patients (*n* = 568)		
		*n*	%	95% CI
Any skin abnormality	*Yes*	85	15.0%	12.3%–18.1%
Morphological skin abnormality	*Yes*	24	4.2%	2.9%–6.2%
Cutis laxa	*Yes*	66	11.6%	9.2%–14.5%
Skin lesion	*Yes*	6	1.1%	0.5%–2.3%
Any deafness	*Yes*	97	17.1%	14.2%–20.4%
Mixed deafness	*Yes*	25	4.4%	3.0%–6.4%
Conductive deafness	*Yes*	32	5.6%	4.0%–7.8%
Sensorineural deafness	*Yes*	36	6.3%	4.6%–8.6%
Other deafness	*Yes*	4	0.7%	0.3%–1.8%
Any valvulopathy	*Yes*	67	11.8%	9.4%–14.7%
Aortic valvulopathy	*Yes*	8	1.4%	0.7%–2.8%
Mitral valvulopathy	*Yes*	43	7.6%	5.7%–10.0%
Pulmonary valvulopathy	*Yes*	4	0.7%	0.3%–1.8%
Tricuspid valvulopathy	*Yes*	15	2.6%	1.6%–4.3%
Sclera	*White*	120	21.1%	18.0%–24.7%
	*Blue*	369	65.0%	61.0%–68.8%
	*Gray/purple*	79	13.9%	11.3%–17.0%
Joint Hyperlaxity	*Yes*	217	38.2%	34.3%–42.3%
Any dental defect	*Yes*	109	19.2%	16.2%–22.6%
Dentinogenesis imperfecta	*Yes*	89	15.7%	12.9%–18.9%
Other dental defects	*Yes*	52	9.2%	7.0%–11.8%

CI, confidence interval.

The presence of valvulopathies was reported in 67 patients (11.8%), and most of the cases involved the mitral valve that allows blood to flow from the left atrium to the left ventricle. The incidence of valvulopathies was higher in female than male patients (14.1% and 9.1%, respectively).

Moreover, 19.2% of the patients presented teeth defects, in terms of DI (15.7%) and other teeth abnormalities (9.2%); in addition, teeth defects were more present in OI type III (45.2%) over type IV (32.2%) and type I (15.8%).

Joint hyperlaxity was reported in 217 (38.2%) patients, with an almost equal distribution among genders (36% in male patients and 40.1% in female patients) and OI types, representing one of the most common extraskeletal manifestations in OI patients.

### 3.4 Inheritance and genetic background

The inheritance pattern was available for just over half of the cohort (53%), and two-thirds of them (35.9%) had a positive familial inheritance.

Genetic variants were identified in 437 patients (76.9%), while in the remaining subjects, the molecular alteration was not detected after extensive DNA analysis, albeit the evident clinical diagnosis of OI. Variants in *COL1A1* and *COL1A2* were the most commonly detected alterations responsible for OI (71.6% and 25.6%, respectively); nevertheless, variants in non-collagen genes (*CRTAP*, *FKBP10*, *IFITM5*, *LEPRE1*, *PLS3*, *SERPINF1*) were found in 2.7% of the patients. Except for *IFITM5*, the other genes are associated with the autosomal recessive forms of OI. Most of the causative variants (98%) are in heterozygosity, and only seven homozygous variants and two compound heterozygosity were identified, all of them on non-collagen genes.

The most frequently detected mutation types were frameshifts and big rearrangements (31.1%) and glycine substitutions (29.5%), followed by splice site variants (17.4%) and nonsense mutations (16.5%). Moreover, eight variants of unknown significance—seven on *COL1A1* and one on *COL1A2*—were detected. Additional information on the genetic characteristics is listed in **Table 5**.

**Table 5:** Inheritance and genetic characteristics.

		All patients (*n* = 568)		Patients with pathogenic variants (*n* = 437)	
		*n*	%	*n*	%
Inheritance	*No*	97	17.1%	72	16.5%
	*Yes*	204	35.9%	122	27.9%
	*Unknown*	267	47.0%	243	55.6%
Mutation	*No*	131	23.1%		
	*Yes*	437	76.9%	437	100.0%
Mutational status	*No mutation*	131	23.1%		
	*Homozygosis/compound heterozygosis*	9	1.6%	9	2.1%
	*Heterozygosis*	428	75.4%	428	97.9%
Mutated gene	*COL1A1*	313	55.1%	313	71.6%
	*COL1A2*	112	19.7%	112	25.6%
	*CRTAP*	1	0.2%	1	0.2%
	*FKBP10*	1	0.2%	1	0.2%
	*IFITM5*	3	0.5%	3	0.7%
	*LEPRE1*	3	0.5%	3	0.7%
	*PLS3*	1	0.2%	1	0.2%
	*SERPINF1*	3	0.5%	3	0.7%
	*Negative for COL1*	109	19.2%		
	*Negative for COL1− and other genes*	22	3.9%		
Dominant mutation	*No mutation*	131	23.1%		
	*No*	9	1.6%	9	2.1%
	*Yes*	428	75.4%	428	97.9%
Effect of mutation	*No mutation or non-COL1 mutation*	143	25.2%	12	2.7%
	*Quantitative/haploinsufficiency*	202	35.6%	202	46.2%
	*Qualitative/structural*	223	39.3%	223	51.0%
Mutation type	*No mutation*	131	23.1%		
	*Splice site*	76	13.4%	76	17.4%
	*Deletion/insertion/duplication/frameshift/big rearrangements*	136	23.9%	136	31.1%
	*Glycine substitution*	129	22.7%	129	29.5%
	*Other missense*	20	3.5%	20	4.6%
	*Nonsense*	72	12.7%	72	16.5%
	*Initiating methionine*	4	0.7%	4	0.9%

Among the 425 patients harboring causative mutations on collagen genes, 39.3% were qualitative variants, leading to the synthesis of structurally abnormal collagens, while 35.6% were quantitative variants, caused by a premature termination codon.

### 3.5 OI type V

Among the entire dataset of 568 patients, just two had been clinically categorized as type V: a 4-year-old female patient with unknown inheritance and a 30-year-old male patient with a negative family history. Despite those differences, the two cases are characterized by the same heterozygous recurrent *IFITM5* variant in the untranslated region (c.-14C>T - p. Met1ext-5) (15). Both patients present white sclera, a relatively low number of fractures (higher in the older patient), lower limb limitations, and trunk deformity (in terms of spine alignment). The 30-year-old male patient also showed a low stature (*z*-score −5.85), while the young girl’s height was in line with her peers (*z*-score 0.37). The skeletal and extraskeletal clinical features were similar in the two cases, despite the large age difference.

## 4 Discussion

The present study describes the largest Italian cohort of patients affected by osteogenesis imperfecta, representing one of the largest ever described. This cohort is characterized by an equal representation of adults and children, giving the opportunity to develop a clear picture of disease traits at skeletal and extraskeletal sites over the entire life span.

### 4.1 Demographics, anthropometrics, and OI types

Despite debate in the literature, the present data are in line with several other large OI cohorts, with a slightly higher prevalence in female than male patients (1, 16). Similarly, the distribution of OI patients to the different OI types shows a very high prevalence of type I cases followed by the other types (1, 17). In addition—as per other studies (18, 19)—approximately a quarter of cases are at present not classified according to the Sillence classification, despite the presence of detailed clinical features and the evaluation of experienced clinicians.

The height *z*-score of the present cohort is in line with literature data, showing a very low stature in OI type III, a low stature in OI type IV, and a slightly low stature in OI type I (1, 10, 19, 20), even if a study reported lower *z*-scores (10). Weight showed a similar trend (19, 21), as well as BMI (22), albeit these features were less evaluated in the OI literature.

### 4.2 Skeletal features

Several clinical traits have been considered in the present study, aimed at properly describing the complex phenotype of OI patients. Among them, a key aspect is the presence of deformities and functional limitations. The percentage of patients affected by deformities and/or limitations is still debated in the literature: Mrosk’s study showed deformities, in terms of bone bowing, in the entire cohort, although the study was conducted on a small cohort of 50 patients (2), while another study presented a lower rate of deformities in comparison with the present cohort and a higher presence of limitations (20). In terms of fracture numbers, the present cohort has a low number in comparison with previous studies, particularly considering vertebral compression fractures (21, 23). The reason for this discrepancy and the slightly different incidence of deformities and limitations are mainly due to the high percentage of children presented in our study, which includes circa 200 cases of children under 9 years of age. Furthermore, in our study cohort, there is a high representation of type I patients. In addition, considering the very large cohort of patients investigated, some very mild OI patients were diagnosed due to the presence of other affected family members.

The evaluation of BMD in terms of osteopenia and osteoporosis shows data in line with the literature both considering overall population and subdividing patients according to OI types (1, 19), highlighting a higher presence of osteoporosis in severe OI types.

The last evaluated skeletal feature is facial dysmorphisms—in terms of maxilla deformity, frontal bossing, triangular face, prognathism, and concave nasal ridge. Those traits are not well investigated in the literature. Some case reports describe very severe facial deformities and related surgical approaches (24, 25); in addition, a cephalometric study investigated, via a standardized radiographic protocol, the presence of facial dysmorphisms in OI patients (26). Despite this, the present cohort shows the presence of facial dysmorphisms in more than one-fifth of the cases.

### 4.3 Extraskeletal features

Osteogenesis imperfecta is frequently characterized by several non-bony manifestations, affecting different organs and systems, impacting the patients’ lives. Evaluating the presence of those features in a large OI population can give useful feedback for clinicians on implementing a diagnostic process and care.

An alteration in the color of the sclera is known to be a very frequent trait of OI patients. In the present cohort, non-white sclera was observed in the majority of patients (80%), with a higher presence of bluish discoloration over a gray one. This finding is in line with other studies, especially for OI type I and type III (1, 19, 21, 23).

The presence of skin abnormalities, mainly represented by loose skin, was captured in one-sixth of the evaluated population. This feature is considered a frequent manifestation in OI patients since collagen molecules are protein components of the extracellular matrix of the skin (9). Nevertheless, until the present study, a clear incidence of this trait has not been defined in large patient cohorts and has been mainly considered in case reports (27).

We observed hearing defects in 17% of our patient cohort, consistent with previous reports (18, 20), highlighting the impact of non-bony OI aspects on patients.

One of the most feared features of OI is the increased risk of heart problems. In fact, the presence of valvulopathies, which are the most commonly reported heart disease (28), was detected in almost 12% of the cohort. Those data are in line with previous literature studies showing a higher risk of heart disease in OI patients in comparison to the general population (17, 29).

Dentinogenesis imperfecta and dental defects have been investigated and debated in the literature with a prevalence ranging from 16% to over 60% (10, 19, 20). We detected this feature in almost one-fifth of the overall cohort, with a higher prevalence of DI over all the other dental defects, which include several dental alterations in terms of color, loss, and frailty, such as fragile teeth, premature loss, anodontia, amelogenesis imperfecta, abnormal dental color, and dentition. Nevertheless, the presence of dental abnormalities is less frequent in OI type I in comparison to other OI types, as per literature studies (10, 19).

Lastly, joint hyperlaxity is still debated in the literature, ranging from 55% to 100% of the patients; this feature, in the present cohort, has a low incidence (almost 40%) in comparison to several previous studies (10, 18).

### 4.4 Inheritance and genetic epidemiology

Considering that inheritance information was available for half of the cohort, most patients have a positive pattern in line with the literature (10), although some studies showed higher values of positive familial history (10, 30).

In a Swedish cohort of OI types I–IV (1), the proband mutation rate reported was almost 80%, while the present study has a slightly lower variant detection rate. Nevertheless, the rate of detection of pathogenic variants ranges from 64% to 100% in medium to large cohorts of OI patients (1, 20, 21, 30, 31). Among the 437 patients with a defined pathogenic variant, most of them were detected on *COL1A1*, followed by *COL1A2* and non-collagen genes. When analyzing the variants by mutation types, we detected several frameshifts, big rearrangements, and glycine substitutions. Taking into consideration patients harboring a collagen variant, the present study reports an almost equal presence of qualitative and quantitative defects. In the literature, the Bardai study showed a distribution in line with our data (19), while other studies reported a higher presence of variants impacting protein sequence in the triple-helical domain, leading to the synthesis of structurally abnormal collagen (1, 10).

### 4.5 Limitations

Although our research reports the spectrum of clinical features and genetic epidemiology of a very large Italian OI cohort and reveals useful information and meaningful data on patients’ phenotype, some limitations need to be discussed. First, despite the accepted role of bisphosphonate and other drugs or supplements for the treatment of OI, the evaluation of pharmacological therapy was not included, due to the scarcity and incompleteness of information. Nonetheless, data on drug treatment are frequently imprecise and difficult to evaluate and compare due to the extreme variability in type, duration, administration route, age at treatment, etc. Second, as per other research studies, some patients are not categorized following the Sillence classification, since some are very young, which makes it difficult to properly assign them to a specific subgroup of OI, while others manifested with a clinical phenotype with borderline aspects between type I and IV or between type III and IV. Lastly, only two patients were diagnosed with OI type V; however, type V is an ultrarare disease subgroup, and the limited number of cases in our cohort is in line with the literature.

### 4.6 Conclusion

In conclusion, this study presents the clinical manifestations of the largest Italian OI patient cohort ever evaluated and their genetic background, giving a clear picture of the heterogeneous phenotype and diverse severity in terms of skeletal and extraskeletal manifestations. A standardized and structured collection of high-quality data, via disease registries, can support and inform clinicians in monitoring the disease and in defining the time and type of follow-up, leading to improved clinical practice and patient care with timely interventions.

## Data availability statement

The raw data supporting the conclusions of this article will be made available by the authors, without undue reservation.

## Author contributions

MM: Conceptualization, Methodology, Project administration, Writing – original draft, Writing – review & editing, Formal analysis, Investigation. MB: Data curation, Writing – review & editing, Formal analysis, Conceptualization, Validation. FB: Writing – original draft, Writing – review & editing, Conceptualization, Data curation, Formal analysis, Validation. DA: Data curation, Writing – review & editing, Investigation. SC: Data curation, Investigation, Writing – review & editing, Methodology. MG: Data curation, Investigation, Writing – review & editing, Conceptualization. MLo: Conceptualization, Data curation, Writing – review & editing, Methodology. EP: Data curation, Methodology, Writing – review & editing, Investigation. ES: Data curation, Investigation, Writing – review & editing. GT: Data curation, Investigation, Writing – review & editing, Methodology. MLa: Data curation, Methodology, Writing – review & editing, Formal analysis. LS: Funding acquisition, Project administration, Resources, Writing – review & editing, Conceptualization.
