# Supplementary material for: Osteogenesis imperfecta: a cross-sectional study of skeletal and extraskeletal features in a large cohort of Italian patients
Source: Front Endocrinol (Lausanne). 2024 Jan 11;14:1299232. doi: 10.3389/fendo.2023.1299232 (PMC10809148; doi:10.3389/fendo.2023.1299232)
Supplement: Supplementary file 1 [file Table_1.docx]

**Table S1. Demographic and anthropometric characteristics of OI patients, by sex**

|  |  |  | **Males (n = 264)** | | **Females (n = 304)** | |
| --- | --- | --- | --- | --- | --- | --- |
| OI Type | *Unknown* | n % | 80 | 30.3% | 86 | 28.3% |
|  | *I* | n % | 141 | 53.4% | 169 | 55.6% |
|  | *III* | n % | 14 | 5.3% | 17 | 5.6% |
|  | *IV* | n % | 28 | 10.6% | 31 | 10.2% |
|  | *V* | n % | 1 | 0.4% | 1 | 0.3% |
| OI inheritance | *No* | n % | 40 | 15.2% | 57 | 18.8% |
|  | *Yes* | n % | 104 | 39.4% | 100 | 32.9% |
|  | *Unknown* | n % | 120 | 45.5% | 147 | 48.4% |
| Age | *Years* | mean sd | 18.61 | 16.79 | 25.38 | 18.69 |
|  | *0-1* | n % | 38 | 14.4% | 25 | 8.2% |
|  | *2-9* | n % | 68 | 25.8% | 67 | 22.0% |
|  | *10-17* | n % | 55 | 20.8% | 33 | 10.9% |
|  | *18-34* | n % | 52 | 19.7% | 82 | 27.0% |
|  | *35-50* | n % | 36 | 13.6% | 64 | 21.1% |
|  | *50-65* | n % | 12 | 4.5% | 23 | 7.6% |
|  | *65-75* | n % | 3 | 1.1% | 10 | 3.3% |
| Height ^a^ | *Z score* | mean sd | -1.54 | 2.23 | -1.47 | 2.20 |
|  | *Percentile* | mean sd | 25.48 | 29.89 | 25.44 | 30.02 |
| Weight ^b^ | *Z score* | mean sd | -0.60 | 1.63 | -0.66 | 1.87 |
|  | *Percentile* | mean sd | 38.92 | 32.82 | 38.91 | 33.71 |
| BMI ^c^ | *Z score* | mean sd | 0.19 | 1.46 | 0.16 | 1.52 |
|  | *Percentile* | mean sd | 56.32 | 32.49 | 55.00 | 34.66 |
|  | *Very Under Weight* | n % | 4 | 2.0% | 6 | 2.5% |
|  | *Under Weight* | n % | 16 | 7.9% | 38 | 15.8% |
|  | *Normal Weight* | n % | 123 | 60.6% | 138 | 57.5% |
|  | *Overweight* | n % | 37 | 18.2% | 35 | 14.6% |
|  | *Obese* | n % | 23 | 11.3% | 23 | 9.6% |

*sd = standard deviation; a = data were missing for 79 (13.9%) patients; b = data were missing for 120 (21.1%) patients; c = data were missing for 125 (22%) patients*

**Table S2. Demographic and anthropometric characteristics of OI patients, by OI Type**

|  |  |  | **OI Type I (n = 310)** | | **OI Type III (n = 31)** | | **OI Type IV (n = 59)** | | **OI Type V (n = 2)** | |
| --- | --- | --- | --- | --- | --- | --- | --- | --- | --- | --- |
| OI inheritance | *No* | n % | 54 | 17.4% | 13 | 41.9% | 12 | 20.3% | 1 | 50.0% |
|  | *Yes* | n % | 112 | 36.1% | 16 | 51.6% | 22 | 37.3% | 1 | 50.0% |
|  | *Unknown* | n % | 144 | 46.5% | 2 | 6.5% | 25 | 42.4% | 0 | 0.0% |
| Gender | *Male* | n % | 141 | 45.5% | 14 | 45.2% | 28 | 47.5% | 1 | 50.0% |
| Age | *Yyears* | mean sd | 21.96 | 17.70 | 18.67 | 15.35 | 22.66 | 18.06 | 17.26 | 18.75 |
|  | *0-1* | n % | 25 | 8.1% | 6 | 19.4% | 10 | 16.9% | 0 | 0.0% |
|  | *2-9* | n % | 81 | 26.1% | 8 | 25.8% | 10 | 16.9% | 1 | 50.0% |
|  | *10-17* | n % | 53 | 17.1% | 1 | 3.2% | 6 | 10.2% | 0 | 0.0% |
|  | *18-34* | n % | 72 | 23.2% | 9 | 29.0% | 23 | 39.0% | 1 | 50.0% |
|  | *35-50* | n % | 57 | 18.4% | 7 | 22.6% | 4 | 6.8% | 0 | 0.0% |
|  | *50-65* | n % | 14 | 4.5% | 0 | 0.0% | 4 | 6.8% | 0 | 0.0% |
|  | *65-75* | n % | 8 | 2.6% | 0 | 0.0% | 2 | 3.4% | 0 | 0.0% |
| Height ^a^ | *Z score* | mean sd | -0.89 | 1.45 | -4.89 | 4.18 | -2.77 | 2.66 | -2.74 | 4.40 |
|  | *Percentile* | mean sd | 30.63 | 30.58 | 9.61 | 20.80 | 12.46 | 25.79 | 32.00 | 45.25 |
| Weight ^b^ | *Z score* | mean sd | -0.26 | 1.36 | -2.66 | 2.31 | -1.17 | 2.29 | -0.80 | 1.20 |
|  | *Percentile* | mean sd | 43.57 | 32.86 | 11.35 | 21.09 | 34.54 | 34.24 | 28.50 | 33.23 |
| BMI ^c^ | *Z score* | mean sd | 0.19 | 1.31 | 0.22 | 1.77 | 0.20 | 2.16 | -0.10 | 4.08 |
|  | *Percentile* | mean sd | 55.18 | 32.37 | 57.00 | 39.92 | 59.29 | 38.08 | 50.00 | 70.71 |
|  | *Very Under Weight* | n % | 3 | 1.2% | 1 | 4.5% | 2 | 4.1% | 1 | 50.0% |
|  | *Under Weight* | n % | 29 | 11.2% | 3 | 13.6% | 8 | 16.3% | 0 | 0.0% |
|  | *Normal Weight* | n % | 164 | 63.6% | 10 | 45.5% | 20 | 40.8% | 0 | 0.0% |
|  | *Overweight* | n % | 38 | 14.7% | 4 | 18.2% | 10 | 20.4% | 0 | 0.0% |
|  | *Obese* | n % | 24 | 9.3% | 4 | 18.2% | 9 | 18.4% | 1 | 50.0% |

*sd = standard deviation; a = data were missing for 79 (13.9%) patients; b = data were missing for 120 (21.1%) patients; c = data were missing for 125 (22%) patients*

**Table S3. Prevalence of deformities and limitations in males**

| **Anatomical site** |  |  | **Males (n = 264)** | | | | | |  |
| --- | --- | --- | --- | --- | --- | --- | --- | --- | --- |
|  | **Deformity** | | | **Limitation** | | | **Deformity or Limitation** | | |
|  | **n** | **%** | **95% CI** | **n** | **%** | **95% CI** | **n** | **%** | **95% CI** |
| Any Deformity or Limitation | 118 | 44.7% | 38.8-50.7% | 6 | 2.3% | 1.0-4.9% | 124 | 47.0% | 41.0-53.0% |
| Head & Neck | 0 | 0.0% | - | 0 | 0.0% | - | 0 | 0.0% | - |
| Upper limbs | 11 | 4.2% | 2.3-7.3% | 2 | 0.8% | 0.2-2.7% | 13 | 4.9% | 2.9-8.2% |
| Arm | 1 | 0.4% | 0.1-2.1% | 0 | 0.0% | - | 1 | 0.4% | 0.1-2.1% |
| Elbow | 3 | 1.1% | 0.4-3.3% | 1 | 0.4% | 0.1-2.1% | 4 | 1.5% | 0.6-3.8% |
| Forearm | 4 | 1.5% | 0.6-3.8% | 1 | 0.4% | 0.1-2.1% | 5 | 1.9% | 0.8-4.4% |
| Hand | 3 | 1.1% | 0.4-3.3% | 0 | 0.0% | - | 3 | 1.1% | 0.4-3.3% |
| Shoulder | 5 | 1.9% | 0.8-4.4% | 0 | 0.0% | - | 5 | 1.9% | 0.8-4.4% |
| Lower limbs | 36 | 13.6% | 10.0-18.3% | 5 | 1.9% | 0.8-4.4% | 41 | 15.5% | 11.7-20.4% |
| Ankle | 3 | 1.1% | 0.4-3.3% | 1 | 0.4% | 0.1-2.1% | 4 | 1.5% | 0.6-3.8% |
| Foot | 18 | 6.8% | 4.4-10.5% | 2 | 0.8% | 0.2-2.7% | 20 | 7.6% | 5.0-11.4% |
| Knee | 13 | 4.9% | 2.9-8.2% | 3 | 1.1% | 0.4-3.3% | 16 | 6.1% | 3.8-9.6% |
| Leg | 9 | 3.4% | 1.8-6.4% | 0 | 0.0% | - | 9 | 3.4% | 1.8-6.4% |
| Thigh | 14 | 5.3% | 3.2-8.7% | 0 | 0.0% | - | 14 | 5.3% | 3.2-8.7% |
| Trunk | 108 | 40.9% | 35.1-46.9% | 0 | 0.0% | - | 108 | 40.9% | 35.1-46.9% |
| Ribs | 32 | 12.1% | 8.7-16.6% | 0 | 0.0% | - | 32 | 12.1% | 8.7-16.6% |
| Spine alignment | 100 | 37.9% | 32.2-43.9% | 0 | 0.0% | - | 100 | 37.9% | 32.2-43.9% |
| Lordosis | 8 | 3.0% | 1.5-5.9% | 0 | 0.0% | - | 8 | 3.0% | 1.5-5.9% |
| Kyphosis | 23 | 8.7% | 5.9-12.7% | 0 | 0.0% | - | 23 | 8.7% | 5.9-12.7% |
| Scoliosis | 64 | 24.2% | 19.5-29.8% | 0 | 0.0% | - | 64 | 24.2% | 19.5-29.8% |
| Other spine problems | 5 | 1.9% | 0.8-4.4% | 0 | 0.0% | - | 5 | 1.9% | 0.8-4.4% |
| Pelvis | 3 | 1.1% | 0.4-3.3% | 3 | 1.1% | 0.4-3.3% | 6 | 2.3% | 1.0-4.9% |

*CI = confidence interval*

**Table S4. Prevalence of deformities and limitations in females**

| **Anatomical site** |  |  | **Females (n = 304)** | | | | | |  |
| --- | --- | --- | --- | --- | --- | --- | --- | --- | --- |
|  | **Deformity** | | | **Limitation** | | | **Deformity or Limitation** | | |
|  | **n** | **%** | **95% CI** | **n** | **%** | **95% CI** | **n** | **%** | **95% CI** |
| Any Deformity or Limitation | 168 | 55.3% | 49.6-60.8% | 9 | 3.0% | 1.6-5.5% | 177 | 58.2% | 52.6-63.6% |
| Head & Neck | 2 | 0.7% | 0.2-2.4% | 0 | 0.0% | - | 2 | 0.7% | 0.2-2.4% |
| Upper limbs | 22 | 7.2% | 4.8-10.7% | 4 | 1.3% | 0.5-3.3% | 26 | 8.6% | 5.9-12.2% |
| Arm | 1 | 0.3% | 0.1-1.8% | 0 | 0.0% | - | 1 | 0.3% | 0.1-1.8% |
| Elbow | 6 | 2.0% | 0.9-4.2% | 3 | 1.0% | 0.3-2.9% | 9 | 3.0% | 1.6-5.5% |
| Forearm | 2 | 0.7% | 0.2-2.4% | 0 | 0.0% | - | 2 | 0.7% | 0.2-2.4% |
| Hand | 17 | 5.6% | 3.5-8.8% | 0 | 0.0% | - | 17 | 5.6% | 3.5-8.8% |
| Shoulder | 6 | 2.0% | 0.9-4.2% | 1 | 0.3% | 0.1-1.8% | 7 | 2.3% | 1.1-4.7% |
| Lower limbs | 52 | 17.1% | 13.3-21.7% | 6 | 2.0% | 0.9-4.2% | 58 | 19.1% | 15.1-23.9% |
| Ankle | 2 | 0.7% | 0.2-2.4% | 0 | 0.0% | - | 2 | 0.7% | 0.2-2.4% |
| Foot | 23 | 7.6% | 5.1-11.1% | 4 | 1.3% | 0.5-3.3% | 27 | 8.9% | 6.2-12.6% |
| Knee | 24 | 7.9% | 5.4-11.5% | 0 | 0.0% | - | 24 | 7.9% | 5.4-11.5% |
| Leg | 22 | 7.2% | 4.8-10.7% | 2 | 0.7% | 0.2-2.4% | 24 | 7.9% | 5.4-11.5% |
| Thigh | 16 | 5.3% | 3.3-8.4% | 1 | 0.3% | 0.1-1.8% | 17 | 5.6% | 3.5-8.8% |
| Trunk | 151 | 49.7% | 44.1-55.3% | 1 | 0.3% | 0.1-1.8% | 152 | 50.0% | 44.4-55.6% |
| Ribs | 31 | 10.2% | 7.3-14.1% | 0 | 0.0% | - | 31 | 10.2% | 7.3-14.1% |
| Spine alignment | 149 | 49.0% | 43.4-54.6% | 0 | 0.0% | - | 149 | 49.0% | 43.4-54.6% |
| Lordosis | 9 | 3.0% | 1.6-5.5% | 0 | 0.0% | - | 9 | 3.0% | 1.6-5.5% |
| Kyphosis | 31 | 10.2% | 7.3-14.1% | 0 | 0.0% | - | 31 | 10.2% | 7.3-14.1% |
| Scoliosis | 98 | 32.2% | 27.2-37.7% | 0 | 0.0% | - | 98 | 32.2% | 27.2-37.7% |
| Other spine problems | 11 | 3.6% | 2.0-6.4% | 1 | 0.3% | 0.1-1.8% | 12 | 3.9% | 2.3-6.8% |
| Pelvis | 12 | 3.9% | 2.3-6.8% | 4 | 1.3% | 0.5-3.3% | 16 | 5.3% | 3.3-8.4% |

*CI = confidence interval*

**Table S5. Prevalence of deformities and limitations in Type I OI patients**

| **Anatomical site** |  |  | **Type I OI (n = 310)** | | | | | |  |
| --- | --- | --- | --- | --- | --- | --- | --- | --- | --- |
|  | **Deformity** | | | **Limitation** | | | **Deformity or Limitation** | | |
|  | **n** | **%** | **95% CI** | **n** | **%** | **95% CI** | **n** | **%** | **95% CI** |
| Any Deformity or Limitation | 147 | 47.4% | 41.9-53% | 5 | 1.6% | 0.7-3.7% | 152 | 49.0% | 43.5-54.6% |
| Head & Neck | 1 | 0.3% | 0.1-1.8% | 0 | 0.0% | - | 1 | 0.3% | 0.1-1.8% |
| Upper limbs | 18 | 5.8% | 3.7-9% | 1 | 0.3% | 0.1-1.8% | 19 | 6.1% | 4-9.4% |
| Arm | 1 | 0.3% | 0.1-1.8% | 0 | 0.0% | - | 1 | 0.3% | 0.1-1.8% |
| Elbow | 4 | 1.3% | 0.5-3.3% | 1 | 0.3% | 0.1-1.8% | 5 | 1.6% | 0.7-3.7% |
| Forearm | 4 | 1.3% | 0.5-3.3% | 0 | 0.0% | - | 4 | 1.3% | 0.5-3.3% |
| Hand | 11 | 3.5% | 2-6.2% | 0 | 0.0% | - | 11 | 3.5% | 2-6.2% |
| Shoulder | 4 | 1.3% | 0.5-3.3% | 0 | 0.0% | - | 4 | 1.3% | 0.5-3.3% |
| Lower limbs | 48 | 15.5% | 11.9-19.9% | 5 | 1.6% | 0.7-3.7% | 53 | 17.1% | 13.3-21.7% |
| Ankle | 1 | 0.3% | 0.1-1.8% | 0 | 0.0% | - | 1 | 0.3% | 0.1-1.8% |
| Foot | 24 | 7.7% | 5.3-11.3% | 4 | 1.3% | 0.5-3.3% | 28 | 9.0% | 6.3-12.7% |
| Knee | 19 | 6.1% | 4-9.4% | 0 | 0.0% | - | 19 | 6.1% | 4-9.4% |
| Leg | 22 | 7.1% | 4.7-10.5% | 0 | 0.0% | - | 22 | 7.1% | 4.7-10.5% |
| Thigh | 13 | 4.2% | 2.5-7% | 1 | 0.3% | 0.1-1.8% | 14 | 4.5% | 2.7-7.4% |
| Trunk | 126 | 40.6% | 35.3-46.2% | 0 | 0.0% | - | 126 | 40.6% | 35.3-46.2% |
| Ribs | 26 | 8.4% | 5.8-12% | 0 | 0.0% | - | 26 | 8.4% | 5.8-12% |
| Spine alignment | 112 | 36.1% | 31-41.6% | 0 | 0.0% | - | 112 | 36.1% | 31-41.6% |
| Lordosis | 8 | 2.6% | 1.3-5% | 0 | 0.0% | - | 8 | 2.6% | 1.3-5% |
| Kyphosis | 27 | 8.7% | 6.1-12.4% | 0 | 0.0% | - | 27 | 8.7% | 6.1-12.4% |
| Scoliosis | 77 | 24.8% | 20.4-29.9% | 0 | 0.0% | - | 77 | 24.8% | 20.4-29.9% |
| Other spine problems | 7 | 2.3% | 1.1-4.6% | 0 | 0.0% | - | 7 | 2.3% | 1.1-4.6% |
| Pelvis | 4 | 1.3% | 0.5-3.3% | 5 | 1.6% | 0.7-3.7% | 9 | 2.9% | 1.5-5.4% |

*CI = confidence interval*

**Table S6. Prevalence of deformities and limitations in in Type III OI patients**

| **Anatomical site** |  |  | **Type III OI (n = 31)** | | | | | |  |
| --- | --- | --- | --- | --- | --- | --- | --- | --- | --- |
|  | **Deformity** | | | **Limitation** | | | **Deformity or Limitation** | | |
|  | **n** | **%** | **95% CI** | **n** | **%** | **95% CI** | **n** | **%** | **95% CI** |
| Any Deformity or Limitation | 27 | 87.1% | 71.1-94.9% | 0 | 0.0% | - | 27 | 87.1% | 71.1-94.9% |
| Head & Neck | 1 | 3.2% | 0.6-16.2% | 0 | 0.0% | - | 1 | 3.2% | 0.6-16.2% |
| Upper limbs | 3 | 9.7% | 3.3-24.9% | 0 | 0.0% | - | 3 | 9.7% | 3.3-24.9% |
| Arm | 0 | 0.0% | - | 0 | 0.0% | - | 0 | 0.0% | - |
| Elbow | 1 | 3.2% | 0.6-16.2% | 0 | 0.0% | - | 1 | 3.2% | 0.6-16.2% |
| Forearm | 0 | 0.0% | - | 0 | 0.0% | - | 0 | 0.0% | - |
| Hand | 1 | 3.2% | 0.6-16.2% | 0 | 0.0% | - | 1 | 3.2% | 0.6-16.2% |
| Shoulder | 1 | 3.2% | 0.6-16.2% | 0 | 0.0% | - | 1 | 3.2% | 0.6-16.2% |
| Lower limbs | 8 | 25.8% | 13.7-43.2% | 0 | 0.0% | - | 8 | 25.8% | 13.7-43.2% |
| Ankle | 0 | 0.0% | - | 0 | 0.0% | - | 0 | 0.0% | - |
| Foot | 2 | 6.5% | 1.8-20.7% | 0 | 0.0% | - | 2 | 6.5% | 1.8-20.7% |
| Knee | 3 | 9.7% | 3.3-24.9% | 0 | 0.0% | - | 3 | 9.7% | 3.3-24.9% |
| Leg | 3 | 9.7% | 3.3-24.9% | 0 | 0.0% | - | 3 | 9.7% | 3.3-24.9% |
| Thigh | 4 | 12.9% | 5.1-28.9% | 0 | 0.0% | - | 4 | 12.9% | 5.1-28.9% |
| Trunk | 26 | 83.9% | 67.4-92.9% | 0 | 0.0% | - | 26 | 83.9% | 67.4-92.9% |
| Ribs | 11 | 35.5% | 21.1-53.1% | 0 | 0.0% | - | 11 | 35.5% | 21.1-53.1% |
| Spine alignment | 23 | 74.2% | 56.8-86.3% | 0 | 0.0% | - | 23 | 74.2% | 56.8-86.3% |
| Lordosis | 1 | 3.2% | 0.6-16.2% | 0 | 0.0% | - | 1 | 3.2% | 0.6-16.2% |
| Kyphosis | 5 | 16.1% | 7.1-32.6% | 0 | 0.0% | - | 5 | 16.1% | 7.1-32.6% |
| Scoliosis | 17 | 54.8% | 37.8-70.8% | 0 | 0.0% | - | 17 | 54.8% | 37.8-70.8% |
| Other spine problems | 2 | 6.5% | 1.8-20.7% | 0 | 0.0% | - | 2 | 6.5% | 1.8-20.7% |
| Pelvis | 3 | 9.7% | 3.3-24.9% | 0 | 0.0% | - | 3 | 9.7% | 3.3-24.9% |

*CI = confidence interval*

**Table S7. Prevalence of deformities and limitations in Type IV OI patients**

| **Anatomical site** |  |  | **Type IV OI (n = 59)** | | | | | |  |
| --- | --- | --- | --- | --- | --- | --- | --- | --- | --- |
|  | **Deformity** | | | **Limitation** | | | **Deformity or Limitation** | | |
|  | **n** | **%** | **95% CI** | **n** | **%** | **95% CI** | **n** | **%** | **95% CI** |
| Any Deformity or Limitation | 40 | 67.8% | 55.1-78.3% | 2 | 3.4% | 0.9-11.5% | 42 | 71.2% | 58.6-81.2% |
| Head &Neck | 0 | 0.0% | - | 0 | 0.0% | - | 0 | 0.0% | - |
| Upper limbs | 2 | 3.4% | 0.9-11.5% | 1 | 1.7% | 0.3-9% | 3 | 5.1% | 1.7-13.9% |
| Arm | 0 | 0.0% | - | 0 | 0.0% | - | 0 | 0.0% | - |
| Elbow | 0 | 0.0% | - | 1 | 1.7% | 0.3-9% | 1 | 1.7% | 0.3-9% |
| Forearm | 0 | 0.0% | - | 0 | 0.0% | - | 0 | 0.0% | - |
| Hand | 0 | 0.0% | - | 0 | 0.0% | - | 0 | 0.0% | - |
| Shoulder | 2 | 3.4% | 0.9-11.5% | 0 | 0.0% | - | 2 | 3.4% | 0.9-11.5% |
| Lower limbs | 11 | 18.6% | 10.7-30.4% | 1 | 1.7% | 0.3-9% | 12 | 20.3% | 12-32.3% |
| Ankle | 2 | 3.4% | 0.9-11.5% | 0 | 0.0% | - | 2 | 3.4% | 0.9-11.5% |
| Foot | 3 | 5.1% | 1.7-13.9% | 1 | 1.7% | 0.3-9% | 4 | 6.8% | 2.7-16.2% |
| Knee | 5 | 8.5% | 3.7-18.4% | 0 | 0.0% | - | 5 | 8.5% | 3.7-18.4% |
| Leg | 4 | 6.8% | 2.7-16.2% | 0 | 0.0% | - | 4 | 6.8% | 2.7-16.2% |
| Thigh | 6 | 10.2% | 4.7-20.5% | 0 | 0.0% | - | 6 | 10.2% | 4.7-20.5% |
| Trunk | 40 | 67.8% | 55.1-78.3% | 0 | 0.0% | - | 40 | 67.8% | 55.1-78.3% |
| Ribs | 12 | 20.3% | 12-32.3% | 0 | 0.0% | - | 12 | 20.3% | 12-32.3% |
| Spine alignment | 38 | 64.4% | 51.7-75.4% | 0 | 0.0% | - | 38 | 64.4% | 51.7-75.4% |
| Lordosis | 3 | 5.1% | 1.7-13.9% | 0 | 0.0% | - | 3 | 5.1% | 1.7-13.9% |
| Kyphosis | 10 | 16.9% | 9.5-28.5% | 0 | 0.0% | - | 10 | 16.9% | 9.5-28.5% |
| Scoliosis | 25 | 42.4% | 30.6-55.1% | 0 | 0.0% | - | 25 | 42.4% | 30.6-55.1% |
| Other spine problems | 2 | 3.4% | 0.9-11.5% | 0 | 0.0% | - | 2 | 3.4% | 0.9-11.5% |
| Pelvis | 3 | 5.1% | 1.7-13.9% | 1 | 1.7% | 0.3-9% | 4 | 6.8% | 2.7-16.2% |

*CI = confidence interval*

**Table S8. Prevalence of deformities and limitations in in Type V OI patients**

| **Anatomical site** |  |  | **Type V OI (n = 2)** | | | | | |  |
| --- | --- | --- | --- | --- | --- | --- | --- | --- | --- |
|  | **Deformity** | | | **Limitation** | | | **Deformity or Limitation** | | |
|  | **n** | **%** | **95% CI** | **n** | **%** | **95% CI** | **n** | **%** | **95% CI** |
| Any Deformity or Limitation | 2 | 100.0% | - | 0 | 0.0% | - | 2 | 100.0% | 34.2-100% |
| Head & Neck | 0 | 0.0% | - | 0 | 0.0% | - | 0 | 0.0% | - |
| Upper limbs | 0 | 0.0% | - | 0 | 0.0% | - | 0 | 0.0% | - |
| Arm | 0 | 0.0% | - | 0 | 0.0% | - | 0 | 0.0% | - |
| Elbow | 0 | 0.0% | - | 0 | 0.0% | - | 0 | 0.0% | - |
| Forearm | 0 | 0.0% | - | 0 | 0.0% | - | 0 | 0.0% | - |
| Hand | 0 | 0.0% | - | 0 | 0.0% | - | 0 | 0.0% | - |
| Shoulder | 0 | 0.0% | - | 0 | 0.0% | - | 0 | 0.0% | - |
| Lower limbs | 0 | 0.0% | - | 1 | 50.0% | 9.5-90.5% | 1 | 50.0% | 9.5-90.5% |
| Ankle | 0 | 0.0% | - | 1 | 50.0% | 9.5-90.5% | 1 | 50.0% | 9.5-90.5% |
| Foot | 0 | 0.0% | - | 0 | 0.0% | - | 0 | 0.0% | - |
| Knee | 0 | 0.0% | - | 1 | 50.0% | 9.5-90.5% | 1 | 50.0% | 9.5-90.5% |
| Leg | 0 | 0.0% | - | 0 | 0.0% | - | 0 | 0.0% | - |
| Thigh | 0 | 0.0% | - | 0 | 0.0% | - | 0 | 0.0% | - |
| Trunk | 2 | 100.0% | - | 0 | 0.0% | - | 2 | 100.0% | - |
| Ribs | 1 | 50.0% | 9.5-90.5% | 0 | 0.0% | - | 1 | 50.0% | 9.5-90.5% |
| Spine alignment | 2 | 100.0% | - | 0 | 0.0% | - | 2 | 100.0% | - |
| Lordosis | 0 | 0.0% | - | 0 | 0.0% | - | 0 | 0.0% | - |
| Kyphosis | 1 | 50.0% | 9.5-90.5% | 0 | 0.0% | - | 1 | 50.0% | 9.5-90.5% |
| Scoliosis | 1 | 50.0% | 9.5-90.5% | 0 | 0.0% | - | 1 | 50.0% | 9.5-90.5% |
| Other spine problems | 0 | 0.0% | - | 0 | 0.0% | - | 0 | 0.0% | - |
| Pelvis | 0 | 0.0% | - | 1 | 50.0% | 9.5-90.5% | 1 | 50.0% | 9.5-90.5% |

*CI = confidence interval*

**Table S9. Prevalence of skeletal features, by sex**

|  |  | **Males**  **(n = 264)** | | | **Females**  **(n = 304)** | | |
| --- | --- | --- | --- | --- | --- | --- | --- |
|  |  | **n** | **%** | **95% CI** | **n** | **%** | **95% CI** |
| Number of fractures | *0* | 31 | 11.7% | 8.4-16.2% | 42 | 13.8% | 10.4-18.1% |
|  | *1-4* | 109 | 41.3% | 35.5-47.3% | 125 | 41.1% | 35.7-46.7% |
|  | *5-10* | 86 | 32.6% | 27.2-38.4% | 85 | 28.0% | 23.2-33.3% |
|  | *11-20* | 20 | 7.6% | 5-11.4% | 36 | 11.8% | 8.7-16% |
|  | *21-30* | 9 | 3.4% | 1.8-6.4% | 9 | 3.0% | 1.6-5.5% |
|  | *>30* | 9 | 3.4% | 1.8-6.4% | 7 | 2.3% | 1.1-4.7% |
| Bone Densitometry | *Osteopenia* | 37 | 14.0% | 10.3-18.7% | 42 | 13.8% | 10.4-18.1% |
|  | *Osteoporosis* | 67 | 25.4% | 20.5-31% | 78 | 25.7% | 21.1-30.8% |
| Any facial dysmorphism | *Yes* | 56 | 21.2% | 16.7-26.5% | 61 | 20.1% | 15.9-24.9% |
| Triangular face | *Yes* | 14 | 5.3% | 3.2-8.7% | 18 | 5.9% | 3.8-9.2% |
| Maxillo dysmorphism | *Yes* | 21 | 8.0% | 5.3-11.9% | 21 | 6.9% | 4.6-10.3% |
| Frontal bossing | *Yes* | 31 | 11.7% | 8.4-16.2% | 29 | 9.5% | 6.7-13.4% |
| Other facial dysmorphism | *Yes* | 3 | 1.1% | 0.4-3.3% | 2 | 0.7% | 0.2-2.4% |
| Wormian Bones | *Yes* | 21 | 8.0% | 5.3-11.9% | 24 | 7.9% | 5.4-11.5% |
| Vertebral Compression Fractures | *Yes* | 5 | 1.9% | 0.8-4.4% | 5 | 1.6% | 0.7-3.8% |

*CI = confidence interval*

**Table S10. Prevalence of skeletal features, in Type I and III OI patients**

|  |  | **Type I OI**  **(n = 310)** | | | **Type III OI**  **(n = 31)** | | |
| --- | --- | --- | --- | --- | --- | --- | --- |
|  |  | **n** | **%** | **95% CI** | **n** | **%** | **95% CI** |
| Number of fractures | *0* | 37 | 11.9% | 8.8-16.0% | 2 | 6.5% | 1.8-20.7% |
|  | *1-4* | 144 | 46.5% | 41.0-52.0% | 11 | 35.5% | 21.1-53.1% |
|  | *5-10* | 96 | 31.0% | 26.1-36.3% | 7 | 22.6% | 11.4-39.8% |
|  | *11-20* | 26 | 8.4% | 5.8-12% | 4 | 12.9% | 5.1-28.9% |
|  | *21-30* | 6 | 1.9% | 0.9-4.2% | 3 | 9.7% | 3.3-24.9% |
|  | *>30* | 1 | 0.3% | 0.1-1.8% | 4 | 12.9% | 5.1-28.9% |
| Bone Densitometry | *Osteopenia* | 52 | 16.8% | 13.0-21.3% | 3 | 9.7% | 3.3-24.9% |
|  | *Osteoporosis* | 76 | 24.5% | 20.1-29.6% | 11 | 35.5% | 21.1-53.1% |
| Any facial dysmorphism | *Yes* | 44 | 14.2% | 10.7-18.5% | 15 | 48.4% | 32.0-65.2% |
| Triangular face | *Yes* | 8 | 2.6% | 1.3-5.0% | 10 | 32.3% | 18.6-49.9% |
| Maxillo dysmorphism | *Yes* | 21 | 6.8% | 4.5-10.1% | 4 | 12.9% | 5.1-28.9% |
| Frontal bossing | *Yes* | 18 | 5.8% | 3.7-9.0% | 4 | 12.9% | 5.1-28.9% |
| Other facial dysmorphism | *Yes* | 2 | 0.6% | 0.2-2.3% | 2 | 6.5% | 1.8-20.7% |
| Wormian Bones | *Yes* | 22 | 7.1% | 4.7-10.5% | 8 | 25.8% | 13.7-43.2% |
| Vertebral Compression Fractures | *Yes* | 3 | 1.0% | 0.3-2.8% | 2 | 6.5% | 1.8-20.7% |

*CI = confidence interval*

**Table S11. Prevalence of skeletal features, in Type IV and V OI patients**

|  |  | **Type IV OI**  **(n = 59)** | | | **Type V OI**  **(n = 2)** | | |
| --- | --- | --- | --- | --- | --- | --- | --- |
|  |  | **n** | **%** | **95% CI** | **n** | **%** | **95% CI** |
| Number of fractures | *0* | 3 | 5.1% | 1.7-13.9% | 0 | 0.0% | - |
|  | *1-4* | 16 | 27.1% | 17.4-39.6% | 1 | 50.0% | 9.5-90.5% |
|  | *5-10* | 21 | 35.6% | 24.6-48.3% | 1 | 50.0% | 9.5-90.5% |
|  | *11-20* | 13 | 22.0% | 13.4-34.1% | 0 | 0.0% | - |
|  | *21-30* | 2 | 3.4% | 0.9-11.5% | 0 | 0.0% | - |
|  | *>30* | 4 | 6.8% | 2.7-16.2% | 0 | 0.0% | - |
| Bone Densitometry | *Osteopenia* | 7 | 11.9% | 5.9-22.5% | 0 | 0.0% | - |
|  | *Osteoporosis* | 24 | 40.7% | 29.1-53.4% | 1 | 50.0% | 9.5-90.5% |
| Any facial dysmorphism | *Yes* | 22 | 37.3% | 26.1-50.0% | 1 | 50.0% | 9.5-90.5% |
| Triangular face | *Yes* | 9 | 15.3% | 8.2-26.5% | 0 | 0.0% | - |
| Maxillo dysmorphism | *Yes* | 4 | 6.8% | 2.7-16.2% | 0 | 0.0% | - |
| Frontal bossing | *Yes* | 14 | 23.7% | 14.7-36.0% | 1 | 50.0% | 9.5-90.5% |
| Other facial dysmorphism | *Yes* | 0 | 0.0% | - | 0 | 0.0% | - |
| Wormian Bones | *Yes* | 8 | 13.6% | 7.0-24.5% | 1 | 50.0% | 9.5-90.5% |
| Vertebral Compression Fractures | *Yes* | 0 | 0.0% | - | 0 | 0.0% | - |

*CI = confidence interval*

**Table S12. Prevalence of extra-skeletal features, by sex**

|  |  | **Males**  **(n = 264)** | | | **Females**  **(n = 304)** | | |
| --- | --- | --- | --- | --- | --- | --- | --- |
|  |  | **n** | **%** | **95% CI** | **n** | **%** | **95% CI** |
| Any skin abnormality | *Yes* | 27 | 10.2% | 7.1-14.5% | 58 | 19.1% | 15.1-23.9% |
| Morphological skin abnormality | *Yes* | 7 | 2.7% | 1.3-5.4% | 17 | 5.6% | 3.5-8.8% |
| Cute laxa | *Yes* | 23 | 8.7% | 5.9-12.7% | 43 | 14.1% | 10.7-18.5% |
| Skin lesion | *Yes* | 0 | 0.0% | - | 6 | 2.0% | 0.9-4.2% |
| Any deafness | *Yes* | 43 | 16.3% | 12.3-21.2% | 54 | 17.8% | 13.9-22.5% |
| Mixed deafness | *Yes* | 12 | 4.5% | 2.6-7.8% | 13 | 4.3% | 2.5-7.2% |
| Conductive deafness | *Yes* | 13 | 4.9% | 2.9-8.2% | 19 | 6.3% | 4.0-9.6% |
| Sensorineural deafness | *Yes* | 17 | 6.4% | 4.1-10.1% | 19 | 6.3% | 4.0-9.6% |
| Other deafness | *Yes* | 1 | 0.4% | 0.1-2.1% | 3 | 1.0% | 0.3-2.9% |
| Any valvulopathy | *Yes* | 24 | 9.1% | 6.2-13.2% | 43 | 14.1% | 10.7-18.5% |
| Aortic valvulopathy | *Yes* | 3 | 1.1% | 0.4-3.3% | 5 | 1.6% | 0.7-3.8% |
| Mitral valvulopathy | *Yes* | 19 | 7.2% | 4.7-11.0% | 24 | 7.9% | 5.4-11.5% |
| Pulmonary valvulopathy | *Yes* | 2 | 0.8% | 0.2-2.7% | 2 | 0.7% | 0.2-2.4% |
| Tricuspid valvulopathy | *Yes* | 2 | 0.8% | 0.2-2.7% | 13 | 4.3% | 2.5-7.2% |
| Sclera | *White* | 68 | 25.8% | 20.9-31.4% | 52 | 17.1% | 13.3-21.7% |
|  | *Blue* | 155 | 58.7% | 52.7-64.5% | 214 | 70.4% | 65.0-75.2% |
|  | *Grey/Purple* | 41 | 15.5% | 11.7-20.4% | 38 | 12.5% | 9.2-16.7% |
| Joint Hyperlaxity | *Yes* | 95 | 36.0% | 30.4-41.9% | 122 | 40.1% | 34.8-45.7% |
| Any dental defect | *Yes* | 42 | 15.9% | 12.0-20.8% | 67 | 22.0% | 17.7-27.0% |
| Dentinogenesis imperfecta | *Yes* | 35 | 13.3% | 9.7-17.9% | 54 | 17.8% | 13.9-22.5% |
| Other dental defect | *Yes* | 19 | 7.2% | 4.7-11.0% | 33 | 10.9% | 7.8-14.9% |

*CI = confidence interval*

**Table S13. Prevalence of extra-skeletal features, in Type I and III OI patients**

|  |  | **Type I OI**  **(n = 310)** | | | **Type III OI**  **(n = 31)** | | |
| --- | --- | --- | --- | --- | --- | --- | --- |
|  |  | **n** | **%** | **95% CI** | **n** | **%** | **95% CI** |
| Any skin abnormality | *Yes* | 47 | 15.2% | 11.6-19.6% | 4 | 12.9% | 5.1-28.9% |
| Morphological skin abnormality | *Yes* | 15 | 4.8% | 3.0-7.8% | 2 | 6.5% | 1.8-20.7% |
| Cute laxa | *Yes* | 37 | 11.9% | 8.8-16.0% | 2 | 6.5% | 1.8-20.7% |
| Skin lesion | *Yes* | 1 | 0.3% | 0.1-1.8% | 0 | 0.0% | - |
| Any deafness | *Yes* | 58 | 18.7% | 14.8-23.4% | 4 | 12.9% | 5.1-28.9% |
| Mixed deafness | *Yes* | 14 | 4.5% | 2.7-7.4% | 2 | 6.5% | 1.8-20.7% |
| Conductive deafness | *Yes* | 22 | 7.1% | 4.7-10.5% | 2 | 6.5% | 1.8-20.7% |
| Sensorineural deafness | *Yes* | 21 | 6.8% | 4.5-10.1% | 0 | 0.0% | - |
| Other deafness | *Yes* | 1 | 0.3% | 0.1-1.8% | 0 | 0.0% | - |
| Any valvulopathy | *Yes* | 32 | 10.3% | 7.4-14.2% | 3 | 9.7% | 3.3-24.9% |
| Aortic valvulopathy | *Yes* | 1 | 0.3% | 0.1-1.8% | 0 | 0.0% | - |
| Mitral valvulopathy | *Yes* | 21 | 6.8% | 4.5-10.1% | 2 | 6.5% | 1.8-20.7% |
| Pulmonary valvulopathy | *Yes* | 2 | 0.6% | 0.2-2.3% | 0 | 0.0% | - |
| Tricuspid valvulopathy | *Yes* | 9 | 2.9% | 1.5-5.4% | 2 | 6.5% | 1.8-20.7% |
| Sclera | *White* | 43 | 13.9% | 10.5-18.2% | 12 | 38.7% | 23.7-56.2% |
|  | *Blue* | 221 | 71.3% | 66.0-76.0% | 13 | 41.9% | 26.4-59.2% |
|  | *Grey/Purple* | 46 | 14.8% | 11.3-19.2% | 6 | 19.4% | 9.2-36.3% |
| Joint Hyperlaxity | *Yes* | 129 | 41.6% | 36.3-47.2% | 12 | 38.7% | 23.7-56.2% |
| Any dental defect | *Yes* | 49 | 15.8% | 12.2-20.3% | 14 | 45.2% | 29.2-62.2% |
| Dentinogenesis imperfecta | *Yes* | 39 | 12.6% | 9.3-16.7% | 13 | 41.9% | 26.4-59.2% |
| Other dental defect | *Yes* | 27 | 8.7% | 6.1-12.4% | 1 | 3.2% | 0.6-16.2% |

*CI = confidence interval*

**Table S14. Prevalence of extra-skeletal features, in Type IV and V OI patients**

|  |  | **Type IV OI**  **(n = 59)** | | | **Type V OI**  **(n = 2)** | | |
| --- | --- | --- | --- | --- | --- | --- | --- |
|  |  | **n** | **%** | **95% CI** | **n** | **%** | **95% CI** |
| Any skin abnormality | *Yes* | 8 | 13.6% | 7.0-24.5% | 1 | 50.0% | 9.5-90.5% |
| Morphological skin abnormality | *Yes* | 2 | 3.4% | 0.9-11.5% | 0 | 0.0% | - |
| Cute laxa | *Yes* | 8 | 13.6% | 7.0-24.5% | 1 | 50.0% | 9.5-90.5% |
| Skin lesion | *Yes* | 0 | 0.0% | - | 0 | 0.0% | - |
| Any deafness | *Yes* | 13 | 22.0% | 13.4-34.1% | 0 | 0.0% | - |
| Mixed deafness | *Yes* | 4 | 6.8% | 2.7-16.2% | 0 | 0.0% | - |
| Conductive deafness | *Yes* | 2 | 3.4% | 0.9-11.5% | 0 | 0.0% | - |
| Sensorineural deafness | *Yes* | 7 | 11.9% | 5.9-22.5% | 0 | 0.0% | - |
| Other deafness | *Yes* | 0 | 0.0% | - | 0 | 0.0% | - |
| Any valvulopathy | *Yes* | 12 | 20.3% | 12.0-32.3% | 0 | 0.0% | - |
| Aortic valvulopathy | *Yes* | 4 | 6.8% | 2.7-16.2% | 0 | 0.0% | - |
| Mitral valvulopathy | *Yes* | 5 | 8.5% | 3.7-18.4% | 0 | 0.0% | - |
| Pulmonary valvulopathy | *Yes* | 1 | 1.7% | 0.3-9.0% | 0 | 0.0% | - |
| Tricuspid valvulopathy | *Yes* | 3 | 5.1% | 1.7-13.9% | 0 | 0.0% | - |
| Sclera | *White* | 14 | 23.7% | 14.7-36.0% | 2 | 100.0% | - |
|  | *Blue* | 34 | 57.6% | 44.9-69.4% | 0 | 0.0% | - |
|  | *Grey/Purple* | 11 | 18.6% | 10.7-30.4% | 0 | 0.0% | - |
| Joint Hyperlaxity | *Yes* | 24 | 40.7% | 29.1-53.4% | 0 | 0.0% | - |
| Any dental defect | *Yes* | 19 | 32.2% | 21.7-44.9% | 0 | 0.0% | - |
| Dentinogenesis imperfecta | *Yes* | 18 | 30.5% | 20.3-43.1% | 0 | 0.0% | - |
| Other dental defect | *Yes* | 9 | 15.3% | 8.2-26.5% | 0 | 0.0% | - |

*CI = confidence interval*

**Table S15. Relationship between OI types and genes harbouring pathogenic variant**

|  |  |  | ***OI Type I (n = 310)*** | | ***OI Type III (n = 31)*** | | ***OI Type IV (n = 59)*** | | ***OI Type V (n = 2)*** | |
| --- | --- | --- | --- | --- | --- | --- | --- | --- | --- | --- |
| *Mutated gene* | *COL1A1* | *n %* | *186* | *60.0%* | *10* | *32.3%* | *30* | *50.8%* | *0* | *0.0%* |
|  | *COL1A2* | *n %* | *52* | *16.8%* | *9* | *29.0%* | *9* | *15.3%* | *0* | *0.0%* |
|  | *CRTAP* | *n %* | *0* | *0.0%* | *0* | *0.0%* | *0* | *0.0%* | *0* | *0.0%* |
|  | *FKBP10* | *n %* | *0* | *0.0%* | *1* | *3.2%* | *0* | *0.0%* | *0* | *0.0%* |
|  | *IFITM5* | *n %* | *0* | *0.0%* | *0* | *0.0%* | *1* | *1.7%* | *2* | *100.0%* |
|  | *LEPRE1* | *n %* | *0* | *0.0%* | *1* | *3.2%* | *0* | *0.0%* | *0* | *0.0%* |
|  | *PLS3* | *n %* | *1* | *0.3%* | *0* | *0.0%* | *0* | *0.0%* | *0* | *0.0%* |
|  | *SERPINF1* | *n %* | *0* | *0.0%* | *0* | *0.0%* | *0* | *0.0%* | *0* | *0.0%* |
|  | *Negative for COL1* | *n %* | *63* | *20.3%* | *5* | *16.1%* | *14* | *23.7%* | *0* | *0.0%* |
|  | *Negative for COL1- & other genes* | *n %* | *8* | *2.6%* | *5* | *16.1%* | *5* | *8.5%* | *0* | *0.0%* |
